# Modulation of GABAergic transmission in development and neurodevelopmental disorders: investigating physiology and pathology to gain therapeutic perspectives

**DOI:** 10.3389/fncel.2014.00119

**Published:** 2014-05-22

**Authors:** Gabriele Deidda, Ignacio F. Bozarth, Laura Cancedda

**Affiliations:** Department of Neuroscience and Brain Technologies, Istituto Italiano di TecnologiaGenova, Italy

**Keywords:** GABA, neurodevelopmental disorders, cation chloride cotransporters, GABAA receptor, GABA metabolism

## Abstract

During mammalian ontogenesis, the neurotransmitter GABA is a fundamental regulator of neuronal networks. In neuronal development, GABAergic signaling regulates neural proliferation, migration, differentiation, and neuronal-network wiring. In the adult, GABA orchestrates the activity of different neuronal cell-types largely interconnected, by powerfully modulating synaptic activity. GABA exerts these functions by binding to chloride-permeable ionotropic GABA_A_ receptors and metabotropic GABA_B_ receptors. According to its functional importance during development, GABA is implicated in a number of neurodevelopmental disorders such as autism, Fragile X, Rett syndrome, Down syndrome, schizophrenia, Tourette's syndrome and neurofibromatosis. The strength and polarity of GABAergic transmission is continuously modulated during physiological, but also pathological conditions. For GABAergic transmission through GABA_A_ receptors, strength regulation is achieved by different mechanisms such as modulation of GABA_A_ receptors themselves, variation of intracellular chloride concentration, and alteration in GABA metabolism. In the never-ending effort to find possible treatments for GABA-related neurological diseases, of great importance would be modulating GABAergic transmission in a safe and possibly physiological way, without the dangers of either silencing network activity or causing epileptic seizures. In this review, we will discuss the different ways to modulate GABAergic transmission normally at work both during physiological and pathological conditions. Our aim is to highlight new research perspectives for therapeutic treatments that reinstate natural and physiological brain functions in neuro-pathological conditions.

## Introduction

Gamma-aminobutyric acid (GABA) is the main inhibitory neurotransmitter in the mature brain by mainly binding to chloride (Cl^−^) and bicarbonate (HCO_3_^−^)-permeable GABA_A_ receptors (GABA_A_R; Mohler, 2007; Hubner and Holthoff, 2013) and metabotropic GABA_B_ receptors. The regulation of neuronal activity by GABA depends on its metabolism, expression and activity of the pre- and postsynaptic receptors, and on the electrochemical gradient of Cl^−^ across the cellular membrane.

GABA is synthesized from glutamate as a substrate by two glutamic acid decarboxylase enzymes (GAD) of different molecular weight: GAD67 and GAD65 (encoded by the *Gad1* and *Gad2* genes, respectively; Pinal and Tobin, 1998). The two isoforms show striking differences in their developmental expression (Kiser et al., 1998; Buddhala et al., 2009), subcellular localization (Dupuy and Houser, 1996; Buddhala et al., 2009), enzymatic activity (Battaglioli et al., 2003; Fenalti et al., 2007), and gene regulation (Feldblum et al., 1993; Pinal and Tobin, 1998; Buddhala et al., 2009). In particular, GAD67 has a cytosolic localization mostly in the neuronal soma, and it provides basal level of GABA synthesis. Conversely, GAD65 is preferentially located in the axonal terminal and it provides additional supply of GABA in condition of metabolic demand (Asada et al., 1997; Kash et al., 1997; Namchuk et al., 1997; Buddhala et al., 2009).

Once synthesized, vesicular GABA transporters (VGATs), which are embedded in presynaptic vesicular membranes, use the electrochemical gradient for H^+^ to shuffle and pack GABA into small synaptic vesicles (Roth et al., 2012). Upon fusion of the synaptic vesicles to the cell membrane due to incoming action potentials, GABA is released in the synaptic cleft where it acts on ionotropic GABA_A_ and GABA_C_, as well as metabotropic GABA_B_ receptors. The magnitude and direction of the ionic current through GABA_A_Rs exquisitely depends on its driving force, defined as the difference between the electrochemical equilibrium potential of Cl^−^ anions (reversal potential, E_Cl_) and the resting membrane potential of the neuron (V_m_). If this difference is positive or negative, there will be a net flux of Cl^−^ anions through the plasma membrane following GABA_A_R opening, and this will result in a change in the membrane potential of the neuron. In particular, the net flux of Cl^−^ anions through GABA_A_R (i.e., toward inside or outside the cell) critically hinges on its intracellular concentration ([Cl^−^]_i_). In neurons, two main chloride cotransporters are responsible for setting [Cl^−^]_i_. The Na^+^/K^+^/Cl^−^ cotransporter NKCC1 (Blaesse et al., 2009), which imports Cl^−^ into the neuron, and the K^+^/Cl^−^ cotransporter KCC2, which exports Cl^−^ out of the neuron (Rivera et al., 1999; Sernagor et al., 2010; Kahle et al., 2013). When E_Cl_ is close to V_m_, GABA will exert its inhibitory action by a shunting inhibitory mechanism. Indeed, the local increase in membrane GABA_A_R conductance will “hold” the neuron at the E_Cl_, reducing the amplitude of subsequent excitatory postsynaptic potentials (following Ohm's law) and thus shunting any excitatory input (Gonzalez-Burgos et al., 2011).

The termination of GABA action at synapses depends on GABA reuptake into nerve terminals and astrocytes by GABA transporters located at the cell membrane (GATs; Lee et al., 2006b). Finally, the catabolism of GABA depends on the action of GABA transaminase enzyme (GABA-T) and succinate semialdehyde dehydrogenase enzyme (SSADH), which convert GABA into intermediates of the Krebs cycle and substrates for new production of glutamate.

During early development, GABA is depolarizing and mostly excitatory due to high [Cl^−^]_i_, and it plays a key role by regulating a number of processes including migration, morphological maturation and differentiation of neurons (Ben-Ari et al., 2007; Wang and Kriegstein, 2009; Ben-Ari et al., 2012). Accordingly, GABAergic signaling has been implicated in a number of neurodevelopmental disorders, such as autism (Tabuchi et al., 2007; Coghlan et al., 2012), Fragile X (Curia et al., 2009; Coghlan et al., 2012), Rett Syndrome (Medrihan et al., 2008; Coghlan et al., 2012), Down Syndrome (Chakrabarti et al., 2010; Costa and Scott-McKean, 2013), Schizophrenia (Lewis et al., 2012), Tourette Syndrome (Kalanithi et al., 2005; Di Cristo, 2007), and Neurofibromatosis type I (Costa et al., 2002; Diggs-Andrews and Gutmann, 2013). Unfortunately, in most cases GABAergic drugs are dangerous because either they silence the neuronal network activity in a developing brain or, more often, they trigger epilepsy in patients with neurodevelopmental disorders, who -similarly to infants- (Connell et al., 1989; Sankar and Painter, 2005; Briggs and Galanopoulou, 2011; Loscher et al., 2013) are at higher risk of seizures *per se* (Rissman and Mobley, 2011; Coghlan et al., 2012; Ostendorf et al., 2013; Williams et al., 2013). Finding viable therapeutic treatments targeting GABAergic transmission in neurodevelopmental disorders is thus both of great importance and a big challenge. Addressing this issue implies the study of the fine modulation of GABAergic transmission during physiological conditions as a starting point to tackle dysfunctional transmission in neurodevelopmental disorders. The overall goal of such a challenging approach is to find a physiologically subtler way to safely modulate GABAergic signaling without hurting the delicate equilibrium of the neuronal-network activity in a developing brain.

In the first part of this review, we will discuss different ways to modulate GABAergic transmission naturally at work during physiological conditions. In the second part, we will discuss how GABAergic transmission is affected in disease, and we will give examples of therapeutic approaches that are currently utilized to reinstate brain functions in neuro-pathological conditions.

## Modulation of GABAergic transmission under normal physiological conditions

Modulation of GABAergic transmission is achieved under different physiological conditions in the brain in diverse ways, ranging from regulation of GABA_A_R expression/activity and GABA metabolism to regulation of intracellular chloride concentration.

### Modulation of GABA_A_ receptor function

GABA_A_Rs are composed of 5 protein subunits belonging to 19 different classes (α_1–6_, β_1–3_, γ_1–3_, δ, ϵ, θ, π, ρ_1–3_; Simon et al., 2004; Luscher et al., 2011; Hubner and Holthoff, 2013; Fritschy and Panzanelli, 2014). The assembly of GABA_A_Rs as heteropentamers produces complex heterogeneity in their structure, which is the major determinant of their pharmacological profile and kinetics (Barnard et al., 1998; Luscher et al., 2011; Fritschy and Panzanelli, 2014). In adult mammals, the most common configuration at the synapse comprises two α_1_, two β_2_, and one γ_2_ subunits. This composition guarantees fast and short-lasting (phasic) inhibition mediated by fast desensitizing postsynaptic receptors. Interestingly, GABA_A_R composition changes during ontogenesis, in order to meet the needs of continuously developing neuronal networks. During early development, when synaptogenesis is not fully established, GABA signaling through GABA_A_Rs is mainly achieved at extra synapticsites. Extrasynaptic GABA_A_Rs contain α_5_ or δ, as well as β_2/3_ and γ subunits, allowing “ambient” GABA to elicit long-lasting (tonic) currents that result in persistent depolarization in young neurons (Cellot and Cherubini, 2013). This tonic activation of extrasynaptic GABA_A_Rs is critical at that time, as it regulates cell migration, proliferation, neurite growth and synapse formation (Ben-Ari et al., 2007; Wang and Kriegstein, 2009). Interestingly, when synaptogenesis begins, α_5_ subunits are downregulated and its neuronal localization switches from the soma to the dendritic compartment in the hippocampus (Ramos et al., 2004).

Upon the establishment of structural synapses, functional communication between neurons requires GABA_A_Rs localized at the synapses. At that time, the first formation of primitive functional neuronal circuits follows the hebbian rule “neurons that fire together wire together” (Ben-Ari et al., 2007). Consequently, GABA_A_R kinetics are determined by the widespread distribution of α_2/3_ subunits, which ensures long-lasting currents well-suited for increasing probability of concurrent spiking from different cells (Fritschy et al., 1994; Uusi-Oukari and Korpi, 2010). As development progresses, α_2_ and α_3_ subunits are downregulated, whereas α_1_ and α_6_ subunits are up-regulated (Laurie et al., 1992; Poulter et al., 1992; Uusi-Oukari and Korpi, 2010). This results in a faster decay time of GABAergic events, which allows precise setting of the temporal window for synaptic integration (Pouille and Scanziani, 2001) and synchronization of neuronal networks (Cobb et al., 1995; Whittington et al., 2011). Of note, intracellular chloride itself has been recently shown to influence the subunit composition, possibly acting as an intracellular messenger (Succol et al., 2012).

Once perinatal development is completed, production of new neurons in the adult (adult neurogenesis) remains restricted only to certain brain niches in the dentate gyrus of the hippocampus and in the olfactory bulb. Interestingly, adult neurogenesis is also under the control of GABAergic transmission (Fritschy and Panzanelli, 2014). Not surprisingly, neurogenesis in early development and in the adult share many similarities. For instance, availability of different GABA_A_R subunits provides a molecular mechanism to precisely control neuronal maturation in the dentate gyrus at spatio-temporal level. Indeed, neuronal progenitors cells (NPCs) express functional GABA_A_Rs composed of α_5_β_3_γ_2_ subunits mediating a tonic response to GABA that maintains NPCs in a quiescence state (Song et al., 2012). Interestingly, mice lacking α_4_ subunits mediating GABA_A_ tonic currents, show increased proliferation and impaired dendritic maturation at early stages. Remarkably, this effect is not present in the mouse line lacking δ subunit, also mediating GABA_A_ tonic currents (Duveau et al., 2011). Conversely, α_2_ subunit, mediating GABA_A_ phasic currents, controls positioning of newborn neurons and dendritic maturation at later stages (Duveau et al., 2011). In the olfactory bulb, specific GABA_A_R subunits also regulate adult neurogenesis, as for example α_2_ subunit regulates neuronal maturation (Pallotto et al., 2012).

Once neuronal circuits are established, and generation of new neurons continues only in discrete niches, the developing brain still retains the potential to learn and respond to experience with plastic changes in neuronal connections. Again, brain plasticity is finely regulated by GABAergic transmission (Fritschy and Panzanelli, 2014). Long-term potentiation (LTP) is a long-lasting increase in the synaptic efficacy elicited by reliable experimental protocols of short, high-frequency stimulation of presynaptic fibers, and it is thus considered as an established model of learning and brain plasticity (Bliss and Collingridge, 1993; Feldman, 2009, 2012). LTP can be easily induced in different regions of the brain (Feldman, 2009, 2012), and in all these structures, GABA_A_R activity prevents or reduces LTP magnitude *in vitro* (Wigstrom and Gustafsson, 1983; Grover and Yan, 1999; Kleschevnikov et al., 2004; Harauzov et al., 2010; Arima-Yoshida et al., 2011; Contestabile et al., 2013) and *in vivo* (Hirai and Okada, 1993; Kaibara and Leung, 1993; Matsuyama et al., 2008; Feldman, 2012). In particular, in the visual cortex, the peculiar contribution of GABA_A_R signaling on LTP depends on the so-called “critical period,” a process of experience-dependent maturation during a specific temporal window after birth. Only during the critical period, but not in the adult, it is possible to induce LTP and another paradigm of plasticity *in vivo* called monocular deprivation (MD), consisting of a functional and an anatomical plastic rearrangement of visual neuronal circuits after a brief period of sight deprivation in one eye (Hensch, 2004; Levelt and Hubener, 2012). Interestingly, GABA controls both the opening and the closure of critical period for plasticity paradigms LTP and MD (Heimel et al., 2011; Levelt and Hubener, 2012). Importantly, critical-period plasticity depends on specific GABA_A_Rs subunits, as demonstrated by the finding that only neuronal circuits containing α_1_ GABA_A_R subunits drive cortical plasticity (Fagiolini et al., 2004; Fritschy and Panzanelli, 2014).

One important mechanism regulating trafficking of GABA_A_Rs as well as their kinetics is phosphorylation of specific α, β, and γ subunits by diverse serine/threonine or tyrosine kinases (Kittler and Moss, 2003; Vithlani et al., 2011). In particular, α_4_ phosphorylation by protein kinase C (PKC) increases insertion and stability of α_4_/β_3_-containing GABA_A_R (Abramian et al., 2010), but also decreases surface expression and activity of α_4_/β_2_/δ containing receptors, possibly suggesting that their differential regulation could be due to the expression of different β subunits (Bright and Smart, 2013). Moreover, phosphorylation of β_1_ subunit by cAMP-dependent kinase (PKA) decreases GABA currents, whereas β_2_ subunit does not respond to PKA (Brandon et al., 2002; Kittler and Moss, 2003). On the other hand, β_2_ subunit can be phosphorylated by Akt increasing postsynaptic GABA_A_R expression (Wang et al., 2003c) and by calcium/calmodulin-dependent protein kinase II (CaMKII) increasing the amplitude of spontaneous inhibitory postsynaptic currents (IPSC; Houston et al., 2008). Conversely, when phosphorylating the β_3_ subunit, CaMKII increases the decay time of sIPSCs (Houston et al., 2008), but also increases surface expression of α_5_/β_3_-containing receptors and tonic currents in different experimental systems (Saliba et al., 2012). Moreover, phosphorylation of the β_3_ subunit by PKA increases GABA currents (McDonald et al., 1998; Brandon et al., 2002). Lastly, phosphorylation of the γ_2_ subunit decreases the internalization rate of GABA_A_Rs and therefore increases their surface expression (Kittler et al., 2008).

Some GABA_A_R subunits are basally phosphorylated (Brandon et al., 2000, 2001), and some phosphatases dephosphorylate specific subunits (Jones and Westbrook, 1997; Huang and Dillon, 1998; Lu et al., 2000; Wang et al., 2003b; Jovanovic et al., 2004; Terunuma et al., 2004; Muir et al., 2010). This suggests that physiological modulation of the phosphorylation state of GABA_A_Rs is finely tuned and may regulate their function. Specifically, protein phosphatases 1α and 2A (but not 2B, i.e., calcineurin) dephosphorylate the β_3_ subunit of the GABA_A_R, leading to decrease of mIPSC amplitude in hippocampal neurons (Jovanovic et al., 2004; Terunuma et al., 2004). On the other hand, NMDA-dependent activation of calcineurin mediates long-term depression (LTD) at inhibitory synapses through γ_2_ subunit dephosphorylation and consequent reduction of GABA_A_R responses (Lu et al., 2000; Wang et al., 2003b). Possibly, GABA_A_R cluster dispersal is responsible for the reduction of GABA_A_R responses, given that NMDA-dependent activation of calcineurin and consequent GABA_A_R dephosphorylation increases lateral diffusion of the receptor itself (Muir et al., 2010).

The efficacy of GABA_A_ergic transmission is also determined by the number of GABA_A_Rs at the synaptic site. As this depends on the rates of insertion and removal of the receptor themselves, endocytosis and rapid recycling (Kittler et al., 2000) or ubiquitin-dependent degradation of GABA_A_Rs (Saliba et al., 2007) can also modify the efficacy of postsynaptic inhibition. Accordingly, changes in lateral diffusion of GABA_A_Rs inside and outside of the synapse, may play a major role in the regulation of GABA_A_ergic synaptic transmission (Triller and Choquet, 2005). Indeed, as described for glutamate receptors (Heine et al., 2008; Petrini et al., 2009), rapid exchange of desensitized receptors between synaptic and extrasynaptic site could allow a higher current to flow at the postsynaptic site.

### Modulation of GABA metabolism

Modulation of GABAergic signaling can also occur at metabolic level, including both anabolism and catabolism of GABA.

GAD enzymes synthetize GABA and they are differentially expressed during ontogenesis, probably reflecting diverse contributions to GABAergic regulation of brain activity. GAD65 and GAD67 are mainly expressed during postnatal development and in the mature brain, whereas two truncated forms of GAD67 (GAD25 and GAD44) are mainly expressed during embryonic development (Buddhala et al., 2009). GAD25 represents the regulatory amino-terminal domain of GAD67, whereas GAD44 represents the enzymatic carboxy-terminal. Although the role of GAD25 and GAD44 during embryonic development has not been understood yet, their expression -at a time when cell proliferation, migration and synaptogenesis occur- has suggested that they could play a role in these processes (Popp et al., 2009). As development progresses, GAD25 and GAD44 expression diminishes accompanied by an increased expression of the active forms of GAD -GAD65 and GAD67- and a concomitant increase of GABA synthesis (Varju et al., 2002).

In particular, GAD67 is expressed earlier than GAD65 (Kiser et al., 1998), it is ubiquitously present in the neuron (Esclapez et al., 1994), and it is expressed as a permanently active enzyme (Lindefors, 1993). Conversely, GAD65 is expressed later than GAD67 (Kiser et al., 1998), it preferentially localizes at synaptic terminals (Esclapez et al., 1994), and it is largely present as an inactive apoenzyme, which can be activated by neuronal activity (Lindefors, 1993; Buddhala et al., 2009). Thus, GAD65 and GAD67 seem to provide a dual system for the control of GABAergic transmission: at earlier stages, GAD67 contributes to tonic GABA transmission necessary for proper development of neuronal circuits (Kaufman et al., 1991; Esclapez et al., 1994). In line with this, GAD67 knockout (KO) mice, which exhibit a 93% reduction of GABA levels, die perinatally (Asada et al., 1997). Once circuits are established (but still immature), GAD65 is expressed and implicated in sustained phasic synaptic transmission in response to neuronal activity (Hensch et al., 1998; Tian et al., 1999; Buddhala et al., 2009). This phasic synaptic transmission supports GABA in reaching a threshold level necessary to render neuronal circuit responsive to experience by functional and anatomical plastic shaping (Fagiolini and Hensch, 2000; Heimel et al., 2011; Levelt and Hubener, 2012). Indeed, GAD65 KO mice are profoundly affected in their ability to respond to monocular deprivation (Hensch et al., 1998).

GABA vesicular transporters (VGAT) mediate transport of GABA into small presynaptic vesicles. Interestingly, absence of VGAT causes embryonic lethality in VGAT KO mice, indicating a fundamental role for GABA release from synaptic vesicles in brain development (Wojcik et al., 2006). Based on the increased expression of VGAT during development, which parallels the developmental switch of GABAergic responses from excitatory to inhibitory, it was proposed that VGAT may also play a role in regulating the latter switch. Nevertheless, the lack of a difference between the expression of KCC2 in VGAT KO in comparison to wild type mice, seems to rule out this hypothesis (Wojcik et al., 2006; Boulland and Chaudhry, 2012). On the other hand, VGAT could still regulate the strength of presynaptic GABA_A_ergic transmission acutely (Kullmann et al., 2005), as *in vitro* experiments show that VGAT co-transports Cl^−^ anions together with GABA (Juge et al., 2009), probably rendering [Cl^−^]_i_ lower in the presynaptic terminal. Finally, VGAT promotes GABAergic transmission also indirectly by promoting GABA synthesis. Indeed, VGAT forms a complex with GAD65 in the nerve terminal, and its absence in the VGAT KO mice results in a loss of GAD65 itself from the presynaptic terminal (Jin et al., 2003; Wojcik et al., 2006).

The control of GABA release represents another key step in the regulation of GABAergic transmission. In this respect, an interesting phenomenon is represented by kainate receptors (KARs), which are expressed at presynaptic terminals of GABAergic interneurons. Activation of these receptors results in a decrease in the amplitude of GABAergic currents and a disinhibition of the postsynaptic neuron (Sihra and Rodriguez-Moreno, 2011). Also extracellular-signal-regulated kinases (ERK) can modulate GABA release during neuronal activity (Cui et al., 2008). Moreover, non-vesicular GABA release (i.e., *via* reversal of GAT-1), contributing to GABA phasic transmission (Wu et al., 2007), can be stimulated by nitric oxide (Tarasenko et al., 2014).

Once GABA is released, GABA transporters at the cell membrane of axon terminals are responsible for GABA removal from the synaptic cleft. GATs modulate GABAergic transmission by regulating the time course of both phasic and tonic GABAergic responses. Even if diffusion of GABA out of the synaptic cleft is the primary determinant for the termination of the response, GABA-uptake inhibitors greatly prolong the phasic conductance associated with application of GABA, pointing to the role of GATs in the termination of GABA-evoked postsynaptic potentials (Dingledine and Korn, 1985; Zhou and Danbolt, 2013). Moreover, GABA uptake becomes of primary importance in conditions of sustained phasic activity of GABAergic neurons (Kersante et al., 2013). On the other hand, GAT regulation is fundamental also for tonic inhibition, as this requires continuous receptor activity elicited by ambient GABA. Indeed, blockade of GABA uptake by GABA transporter blocker NO711 unmasks tonic inhibition in pyramidal neurons in the hippocampus (Semyanov et al., 2003; Walker and Semyanov, 2008). Thus, far, four GATs (GAT-1, GAT-2, GAT-3, BGT-1) have been isolated (Roth and Draguhn, 2012; Zhou and Danbolt, 2013). As for the different GAD isoforms, GATs are differently expressed during development, possibly reflecting diverse needs of dynamically developing neuronal networks. For example, during development, GAT-3 is the mostly expressed in neurons, GAT-2 is poorly expressed, and GAT-1 expression increases gradually (Minelli et al., 1995; Evans et al., 1996; Zhou and Danbolt, 2013). As a result, GAT-1 is the most abundant GAT isoform expressed in neuronal and in astrocytic terminals and GAT-3 is expressed only in astrocytes in the adult cortex (Evans et al., 1996). How the expression of different GATs at diverse time of development and in different cell types may influence GABAergic transmission still needs to be elucidated.

### Regulation of cation/chloride cotransporters

Modulation of GABAergic transmission by variation in [Cl^−^]_i_ is a mechanism naturally at work under a number or circumstances. Interestingly, as Cl^−^ reversal potential (E_Cl_) rests very close to the resting membrane potential of the neuron (V_m_), even small changes in [Cl^−^]_i_ can result in significant shifts of the E_Cl_, for instance from hyperpolarizing to depolarizing. The most characterized example of modulation of GABAergic transmission by variations in [Cl^−^]_i_ occurs during ontogenesis. Indeed, a progressive decrease of [Cl^−^]_i_ is responsible for the shift of the polarity of GABAergic transmission from depolarizing and mostly excitatory during early development to hyperpolarizing and inhibitory in the adult. This developmental shift of GABAergic signaling has been evolutionarily conserved from reptiles to humans, and it possibly provides excitation in the developing brain without the risk of glutamatergic excitotoxicity (Ben-Ari, 2002). Interestingly, this switch also occurs during adult neurogenesis in the dentate gyrus of the hippocampus and in the olfactory bulb (Wang et al., 2003a; Ge et al., 2006, 2008; Sernagor et al., 2010; Young et al., 2010; Ming and Song, 2011; Young et al., 2012).

The higher [Cl^−^]_i_ in young neurons depends on high expression of NKCC1 (Dzhala et al., 2005), and it results in depolarizing GABA_A_ transmission. As development proceeds, KCC2 becomes expressed at higher levels than NKCC1 (Rivera et al., 1999; Dzhala et al., 2005), and hyperpolarizing GABAergic transmission takes over (Ben-Ari, 2002; Ben-Ari et al., 2007; Cancedda et al., 2007; Wang and Kriegstein, 2009; Sernagor et al., 2010). Of note, the functional maturation of GABA_A_R signaling from depolarizing to hyperpolarizing occurs earlier in females than in males (Galanopoulou, 2008), mostly due to the different sex-related expression of NKCC1 and KCC2 (Nunez and McCarthy, 2007).

Another example of how plasticity of E_Cl_ controls GABAergic transmission and brain function under particular physiological circumstances is in the regulation of the circadian rhythm. Mammals, as many other species, entrain their behavior and hormonal daily oscillations to the internal biological clock, which is hierarchically mastered by neurons in the suprachiasmatic nucleus (SCN) of the hypothalamus (Lehman et al., 1987). Interestingly, E_Cl_ undergoes changes during the circadian rhythm in the SCN, although the effective polarity of GABA_A_ergic transmission is still controversial. Indeed, while first studies suggested GABA to be excitatory at night and inhibitory during the day (Liou and Albers, 1990; Bos and Mirmiran, 1993), a later study revealed opposite actions (Wagner et al., 1997). Possibly, the discrepancy between the two sets of results originated from the use of diverse experimental conditions during the extracellular-spike recordings (i.e., standard ACSF extracellular solution vs. solutions modified in chloride concentration) to assess the polarity of GABA_A_ responses. To resolve this controversy, De Jeu and coworkers directly measured E_Cl_ by means of gramicidin-perforated patch-clamp recordings, which allow preservation of [Cl^−^]_i_ and, consequently, a correct assessment of the polarity of GABA_A_ responses (De Jeu and Pennartz, 2002). They found a significant day vs. night difference in E_Cl_ with more depolarized values during the night than during the day. In line with this, successive studies showed that NKCC1 expression was higher at night (Choi et al., 2008). Importantly, the excitatory effect of GABA was reduced by application of NKCC1-inhibitor and loop diuretic bumetanide in brain slices, and absent in *Nkcc1* knock-out mice. This points to a regulation of NKCC1 expression to achieve plasticity of E_Cl_ during the circadian rhythm. This mechanism was proposed to counteract propagation of excitatory signals during the day (Choi et al., 2008).

Another example of modulation of GABA_A_ergic transmission through cation/chloride cotransporters during a particular physiological condition is the shift in E_Cl_ during labor. Birth is a stressful event associated with high risk of injury for the fetal brain, and hypoxic-ischemic brain damage is one of the principal causes of death and neurological impairment in infants (Volpe, 2008). Neuronal activity requires a high metabolic demand since action-potential generation and propagation consumes 47% of available ATP in neurons, while postsynaptic potentials consume another 34% (Attwell and Laughlin, 2001). Consequently, as neuronal activity cannot be easily sustained in condition of hypoxia, the presence of a depolarizing action of GABAergic transmission during delivery could further limit availability of energy supply caused by delivery-induced hypoxia, as experiments on high neuronal activity during seizures would suggest (Dzhala et al., 2000). In a very interesting study, Tyzio and co-workers investigated whether activity of cation/chloride cotransporters during delivery may be altered (Tyzio et al., 2006). They found a shift of E_Cl_ to hyperpolarizing GABA responses 1–2 h before delivery. Interestingly, authors also found that bath application of oxytocin, which is massively released before delivery to induce parturition (Gimpl and Fahrenholz, 2001), suppressed GABA-mediated excitation and induced a negative shift in E_Cl_ in slices from animals 2 days before delivery. Finally, authors demonstrated that oxytocin-mediated effect occurred *via* inhibition of NKCC1 chloride cotransporter by treatment with bumetanide (Tyzio et al., 2006).

Delivery is also potentially painful for the newborn (Fitzgerald, 2005), possibly leading to long-term pathological consequences (Volpe, 2008, 2009). In a follow up study, Mazzuca and coworkers demonstrated that oxytocin exerts an analgesic action against pain in newborn rat pups, assessed in terms of thermal tail-flick test and pain-induced vocalization. Also in this case, the effect of oxytocin was achieved by a reduction of GABA depolarization *via* inhibition of NKCC1, as bumetanide recapitulated the analgesic effects of oxytocin (Mazzuca et al., 2011). These findings are in line with studies in humans indicating that infants born by elective caesarean (c-) section show a lower threshold for pain in comparison to infants born by natural vaginal delivery (Bergqvist et al., 2009), possibly due to the lack of release of maternal oxytocin during delivery by c-section (Gimpl and Fahrenholz, 2001).

The subtle modulation of GABAergic transmission through cation/chloride cotransporters in condition of limiting energy supply finds another example in the animal kingdom (Tyzio et al., 2006). As mentioned above, neuronal activity requires a high metabolic demand and it cannot be easily sustained in condition of hypoxia (Attwell and Laughlin, 2001). The pond snail *Lymnae stagnalis* is a bimodal breather that can use cutaneous gas exchange under well-oxygenated water, and lung exchange in the air atmosphere in hypoxic condition, which naturally encounters during summer time when the pond water where it lives dries out (Jones, 1961). Remarkably, the pond snail is able to survive under harsh hypoxic experimental conditions such as 40 h in a N_2_-bubbled environment (Inoue et al., 2001). This is due to a decrease in neuronal activity mediated by a switch in GABA polarity under hypoxic conditions, and it promotes survival by reducing the energetic demand of the brain (Cheung et al., 2006). Indeed, in normal conditions, GABA exerts an excitatory action in the dorsal ganglia of the pond snail, which helps in maintaining basal levels of spontaneous neuronal activity. Conversely, under hypoxic condition, neurons become less active, an effect mimicked by inhibition of NKCC1 activity with bath application of bumetanide.

Modulation of GABAergic transmission by regulation of cation/chloride cotransporters also occurs at synaptic level. For example, an activity-induced depolarizing shift in E_Cl_ has been described after repetitive postsynaptic spiking (Fiumelli et al., 2005), tetanic stimulation (Kaila et al., 1997), correlated synaptic activity (Woodin et al., 2003), and rebound burst activity (Wang et al., 2006a). Moreover, some evidence also suggests a role for cation/chloride cotransporters in LTP, although with contrasting results. In particular, while some authors report that following high-frequency stimulation and LTP induction there is a downregulation of KCC2, pointing to a more depolarized E_Cl_ (Wang et al., 2006b), other studies indicate that LTP induction results in a shift in E_Cl_ toward more hyperpolarized values (Xu and Sastry, 2007). Of note, in one study, authors found that chloride channel CLC-3 contributes to reducing LTP magnitude, as CLC-3 KO mice show an excessive induction of LTP (Farmer et al., 2013).

In agreement with the studies described above, in the presence of increased neuronal activity driven by physiological sensory stimulation, the brain responds also modulating the plasticity of E_Cl_ by changes in cation/chloride cotransporters. For example, environmental enrichment (EE), which is a combination of inanimate and social stimuli providing a complex sensory-motor stimulation (Rosenzweig et al., 1978; Sale et al., 2014), promotes a precocious maturation of GABAergic transmission in the visual system (Cancedda et al., 2004; Heimel et al., 2011; Levelt and Hubener, 2012). Interestingly, this precocious maturation is accompanied by an accelerated transition of GABA action from depolarizing to hyperpolarizing, possibly due to a concomitant increased expression of KCC2 (He et al., 2010). Finally, recent studies reveal that also maternal deprivation can affect E_Cl_, determining a negative shift of GABA responses. This effect seems to be mediated by a higher expression of KCC2 cotransporter (Galanopoulou, 2008). Thus, the plasticity of E_Cl_ plays a major role in regulating naturally relevant behaviors.

## Modulation of GABAergic transmission in pathology and therapeutic perspectives

Given the pivotal role of GABA in the regulation of brain development and functioning, it is not surprising that alterations in the GABAergic system have been implicated in a number of neurodevelopmental disorders (Di Cristo et al., 2011). As we described above for the modulation of GABAergic signaling in physiology, the alterations of the GABAergic system in neurodevelopmental disorders can occur at the level of GABA receptors, metabolism and/or cation/chloride cotransporters (Table 1).

**Table 1:** **Summary of GABA_A_ signaling alterations in neurodevelopmental disorders**.

**Disorder**	**Main animal model(s)**	**GABA transmission deficits and therapeutic approaches (animal models)**	**GABA transmission deficits (human patients)**	**Selected references**
Autism spectrum disorders	15q11–13 duplication mice	Increased gene expression of α_5,_ β_3,_ and γ_3_ subunits	Dysregulation of interneuron genes	Fatemi et al., 2009, 2010; Liu et al., 2009; Nakatani et al., 2009; Brooks-Kayal, 2010; Penagarikano et al., 2011; Lemonnier et al., 2012; Hadjikhani et al., 2013; Han et al., 2014; Tyzio et al., 2014
	CNTNAP2 KO mice	Decreased GABAergic interneurons	GABA_A_ receptor expression abnormalities (usually downregulation)	
		Presence of seizures		
	BTBR *T*^+^ *Itpr3^tf^*/J mice	Behavioral abnormalities rescued by GABA_A_ α_2_/α_2_ agonists	Higher epilepsy rates	
	*In utero* valproate exposed rats	Bumetanide treatment restores aberrant electrophysiology and vocalizations	Improvement of autistic scores after bumetanide treatment	
			Planned clinical trials with GABA_A_ agonist AZ7325 (http://clinicaltrials.gov/; NCT01966679)	
Fragile X	Fmr1 KO mice	Decreased interneurons and GABA_A_ receptor gene expression	Increased prevalence of epilepsy	D'Hulst et al., 2006; Selby et al., 2007; Olmos-Serrano et al., 2010, 2011; Heulens et al., 2012; Lemonnier et al., 2013; Tyzio et al., 2014
		Hyper excitability in slices and behavior including increased seizure susceptibility which can all be rescued by GABA_A_ positive modulators	Improvement in autistic and intellectual scores after bumetanide treatment	
		Depolarizing GABAergic response rescued by bumetanide	GABA_A_ allosteric modulator Ganaxolone currently in clinical trials (http://clinicaltrials.gov/; NCT01725152)	
Rett syndrome	MECP2 KO mice	Decrease in GAD65 and abnormal interneuron-related gene expression	GABA_A_ receptor dysregulation	Yamashita et al., 1998; Blue et al., 1999; Jian et al., 2006; Medrihan et al., 2008; Voituron and Hilaire, 2011; Durand et al., 2012; Jin et al., 2013
			Epilepsy in 80% of	
		Decreased inhibition in slices	Breathing abnormalities patients	
		Respiratory alterations that are rescued by GABA agonists		
Down syndrome	Ts65Dn mice	Increased number of interneurons	Decreased GABA levels in fetuses and in children	Pueschel et al., 1991; Fernandez et al., 2007; Whittle et al., 2007; Smigielska-Kuzia et al., 2010; Westmark et al., 2010; Chakrabarti et al., 2011; Rissman and Mobley, 2011; Kleschevnikov et al., 2012; Martinez-Cue et al., 2013
		Increased GABAergic signaling, GAD65, GAD67, VGAT all rescued by negative GABA_A_ modulation	Increased seizure susceptibility	
		Decreased LTP and cognitive behavioral tasks that can be rescued by GABA_A_ antagonists	GABA_A_ α_5_ inverse agonist currently in clinical trials (http://clinicaltrials.gov/; NCT01436955,NCT02024789)	
		Increased seizure susceptibility		
Schizophrenia	Non-genetic models:	Decreased GAD65 and GAD67, GAT1 and α, β_3_, δ subunit mRNA	Deficits in GAD65 and GAD67 as well as in reelin, interneurons, GAT1 and multiple GABA_A_ receptor subunits	Wolkowitz and Pickar, 1991; Pierri et al., 1999; Guidotti et al., 2000; Cotter et al., 2002; Harte et al., 2007; Hikida et al., 2007; Hashimoto et al., 2008; Bullock et al., 2009; Cassidy et al., 2010;
	PCP exposition			
	Chronic dopamine (D2) receptor stimulation	Decreased reelin and interneurons		
	Social isolation	Decreased pre-pulse inhibition	Some patients respond well to benzodiazepine treatment	Beneyto et al., 2011; Schmidt and Mirnics, 2012; Del Pino et al., 2013; Yu et al., 2013
	Genetic models:	Decreased parvalbumin cells		
	DISC1 downregulation	Decreased pre-pulse inhibition		
	Erbb4 downregulation			
	GAT1KO			
Tourette syndrome	Striatum injections of GABA_A_ antagonists in rodents and primates	Induction of motor tics resembling those of patients	Interneuron dysregulation	Kalanithi et al., 2005; McCairn et al., 2009; Shprecher and Kurlan, 2009; Worbe et al., 2009; Kataoka et al., 2010; Martinez-Granero et al., 2010; Lerner et al., 2012; Bronfeld et al., 2013; Williams et al., 2013
			Abnormal GABA_A_ receptor binding	
			Increased epilepsy	
			Modest tic suppression by GABA acting drugs	
Neurofibro matosis type 1	NF1 KO mice	Increased GABAergic release and transmission	Decreased GABA levels	Costa and Silva, 2002; Costa et al., 2002; Cui et al., 2008; Ostendorf et al., 2013; Violante et al., 2013
			Increased epilepsy	
		Deficits in memory and LTP that can be rescued by GABA_A_ antagonists		

### Autism spectrum disorders

Autism spectrum disorders (ASDs) are a group of syndromes of diverse etiology with a common set of core symptoms that include compromised social interaction, impaired communication, and repetitive behaviors (Pizzarelli and Cherubini, 2011). ASDs can be idiopathic or co-morbid with other syndromes such as Fragile X or Rett syndrome. Notwithstanding the wide variation in the etiology, the consistency in symptomatology suggests that the mechanisms underlying the pathology of ASDs are common. Mounting evidence indicates that one such mechanism is an impairment in the neuronal excitatory/inhibitory balance (Pizzarelli and Cherubini, 2011), as also suggested by the high prevalence of epilepsy in patients with ASDs (Brooks-Kayal, 2010). Accordingly, several studies have identified mutations in different GABA_A_R subunits as risk factors for the development of ASDs (Coghlan et al., 2012) and autism-related genes have been found to be mostly expressed in interneurons (Xu et al., 2014). In particular, GABRB3, GABRA5, and GABRG3 genes were described as risk factors (Cook et al., 1998; Menold et al., 2001; Buxbaum et al., 2002; Shao et al., 2003; McCauley et al., 2004), and GABRA4 was found to be involved in ASD on its own, but also through interaction with GABRB1 (Ma et al., 2005). Moreover, GABA_A_R subunits α_1–5_, β_1_ and β_3_ and GAD65 and 67 are downregulated in autistic brains (Fatemi et al., 2002, 2009, 2010; Mendez et al., 2013). Accordingly, GABA levels are decreased in the frontal lobe and in the auditory cortex of autistic patients (Harada et al., 2011; Rojas et al., 2013). In addition, mutations in interneuron-associated genes DLX1 and DLX2 have been described as susceptibility factors for ASD. Finally, benzodiazepine binding to GABA_A_Rs is decreased in the hippocampus and frontal cortex of ASD patients (Blatt et al., 2001; Guptill et al., 2007; Liu et al., 2009; Mori et al., 2012).

Benzodiazepines have been used to treat autistic patients along with other antiepileptic drugs (AEDs), mostly because of the comorbidity of ASD and epilepsy. Interestingly, it was proposed that antiepileptics might have an effect on modulating behavioral aspects, as in some studies antiepileptic drugs improved socialization and communication skills (Di Martino and Tuchman, 2001). Nevertheless, consensus on this issue has not been reached yet. In fact, a study by Marrosu and coworkers showed that the use of diazepam had a paradoxical effect by increasing anxiety and aggression in autistic children (Marrosu et al., 1987). This observation led to the hypothesis that GABA could be having excitatory actions in ASD, resulting in a groundbreaking pilot study to test the therapeutical actions of chronic bumetanide treatment in ASD children. By inhibition of NKCC1, the treatment was designed to revert the polarity of GABAergic response from possibly depolarizing to hyperpolarizing in autistic children (Lemonnier and Ben-Ari, 2010). The reduction in autistic scores and the absence of side effects over 3 months of treatment in 5 autistic children led the research group to perform a larger and double-blind clinical trial with 54 patients (Lemonnier et al., 2012). After 3 months of treatment, patients showed a significant amelioration of their symptoms. In addition, this effect was still noticeable (although to a lesser degree) after a 1 month wash-out period. Finally, the same group has recently showed that adolescents and young adults show improvement in emotion recognition after 10 months of treatment with bumetanide (Hadjikhani et al., 2013). Interestingly, an abolished GABA excitatory-inhibitory shift during delivery leading to a chronic deficient chloride regulation and autistic-like behaviors has been recently shown in the valproate and fragile X rodent models of autism (Tyzio et al., 2014). Of note, a planned clinical trial with a selective α_2/3_ selective agonist (AZ7325) will recruit patients soon (http://clinicaltrials.gov/; NCT01966679).

Reinforcing the hypothesis of an involvement of faulty GABAergic transmission in autism, mice with 15q11-13 duplication and contactin-associated protein-like 2 (CNTNAP2) KO mice -two mutations present in some autistic patients- present autistic-like behaviors associated with increased α_5,_ β_3,_ γ_3_ subunit mRNAs as well as decreased number of parvalbumin, calretinin and neuropeptid Y interneurons, respectively (Nakatani et al., 2009; Penagarikano et al., 2011). Further correlation between compromised GABAergic transmission and ASD was found in a mouse mutant of voltage-gated ion channels where the mutation was restricted to forebrain interneurons. These animals exhibited autistic behaviors (i.e., decreased social interaction) that were rescued by benzodiazepine treatment (Han et al., 2012). Moreover, in the BTBR *T*^+^ *Itpr3^tf^*/J (a mouse strain that is used as a model of idiopathic autism given its behavioral profile including poor social interaction) low doses of a drug acting on α_2/3_ subunits (i.e., L-838,417) ameliorate short- and long-term memory as well as social interaction deficits (Han et al., 2014). Finally, the NHE9 gene which encodes a (Na^+^, K^+^)/H^+^ exchanger, is a genetic factor that increases risk for ASD and could potentially affect GABA activity by altering ionic gradients and consequent [Cl^−^]_i_ (Morrow et al., 2008).

### Fragile X syndrome

Fragile X is a monogenic disorder caused by mutations in the FMR1 gene (Bagni and Greenough, 2005). This gene encodes for the Fragile X mental retardation protein (FMRP), which regulates the translation of a wide array of mRNAs. As a considerable amount of Fragile X patients show autistic symptoms and/or comorbidity with epilepsy, neuronal excitatory/inhibitory imbalance has been hypothesized also in these patients (Coghlan et al., 2012). Accordingly, hyper excitability in slices has been described in the Fmr1 KO mouse model (Gibson et al., 2008; Olmos-Serrano et al., 2010; Paluszkiewicz et al., 2011; Goncalves et al., 2013). In particular, recordings in these mice showed decreased sIPSCs and mIPSCs as well as decreased tonic inhibition in the amygdala, and increased firing rate and synchrony in the cortex (Olmos-Serrano et al., 2010; Goncalves et al., 2013 but also see Centonze et al., 2008). Interestingly, this hyperexcitability at synaptic level was accompanied by hyperexcitability at behavioral level, as demonstrated by increased activity in the open field. This could be rescued by GABA_A_ agonist THIP -which increases tonic currents by mainly acting on the δ subunit- at doses that did not have an effect on wild-type mice (Olmos-Serrano et al., 2011).

Among possible causes for the neuronal excitatory/inhibitory imbalance in Fragile X, deficits at different levels of the GABAergic signaling have been described. In particular, decreased density of PV-expressing interneurons was described in Fmr1 KO (Selby et al., 2007). Moreover, deficits in GABA_A_R mRNA and protein levels for different subunits (i.e., α_1_, α_3_, α_4_, β_1_, β_2_, δ, γ_1_, and γ_2_) were reported in Fmr1 KO, although some authors found these defects only during development (El Idrissi et al., 2005; D'Hulst et al., 2006; Gantois et al., 2006; Adusei et al., 2010). Besides downregulation of GABA_A_R subunits, also defects in GABA metabolism have been described, as mRNA for GAD1 and protein expression of GAD65/67 was decreased in Fmr1 KO mice (D'Hulst et al., 2009; Olmos-Serrano et al., 2010), although these studies contrast with others that found GAD65/67 to be increased in these mice (El Idrissi et al., 2005; Adusei et al., 2010). Moreover, mRNA for GAT1 and 4, succinate semialdehyde dehydrogenase (SSADH) and GABA-T are decreased whereas GABA release probability is increased in Fmr1 KO mice (Centonze et al., 2008). Finally, gephyrin (a GABAergic postsynaptic marker) expression is decreased in the cortex of these mice (D'Hulst et al., 2009).

In line with the above findings hypothesizing a decrease in GABAergic transmission in Fragile X, a number of *in vivo* studies attempted to rescue behavioral deficits in mouse models by increasing GABAergic signaling. For example, administration of GABA_A_ agonist taurine rescued performance in the passive avoidance task in Fmr1 KO mice (El Idrissi et al., 2009). Moreover, hyperexcitability in slices and behavior in mice were rescued by the agonist THIP (Olmos-Serrano et al., 2010, 2011). Recently, treatment with diazepam or ganaxolone (a neurostreroid that can positively modulate GABA_A_Rs) rescued audiogenic-induced seizures in Fmr1 KO animals (Heulens et al., 2012). The fact that the δ subunit is downregulated in Fmr1 KO mice and the encouraging results with THIP and ganaxolone -both of which act through the δ subunit- suggest that the δ subunit of the GABA_A_R could be a good therapeutic target in Fragile X. Indeed, ganaxolone is being tested in a Phase 2 clinical trial for children with Fragile X (NCT01725152, http://clinicaltrials.gov/).

Finally, given their earlier success in treating autistic children with bumetanide (Lemonnier et al., 2012), as well as the high incidence of autism in Fragile X patients, Lemonnier and colleagues used bumetanide to treat a child with Fragile X and concomitant autistic diagnosis (Lemonnier et al., 2013). Interestingly, after 3 months of treatment, the child achieved an improvement in several clinical tests addressing autistic behavior and intellectual disability. As this is a promising single case report, further studies will have to evaluate the therapeutic potential of bumetanide treatment for Fragile X on a larger cohort of patients. This is in line with recent articles reporting that switch of GABAergic transmission from depolarizing to hyperpolarizing is delayed in Fmr1KO mice (He et al., 2014). Nevertheless, future treatments of Fragile X adults with bumetanide, may require further pre-clinical investigation because excitatory GABAergic transmission seems to persist in the adult hippocampus whereas inhibitory GABAergic transmission takes eventually over in the cortex of Fmr1KO mice (He et al., 2014; Tyzio et al., 2014).

### Rett syndrome

Rett syndrome is a chromosome X-linked neurodevelopmental disorder caused by mutations in the Methyl-CpG-binding protein 2 (MECP2) gene. Rett syndrome patients develop normally until the age of 6–18 months, but then they undergo a developmental regression that courses with loss of speech, motor coordination, breathing abnormalities and cognitive impairment (Amir et al., 1999; Leblanc and Fagiolini, 2011). Also, epilepsy is present in 80% of patients (Jian et al., 2006). As for the other neurodevelopmental disorders described above, a number of evidence in Rett syndrome patients point to alteration of GABAergic transmission. Indeed, density of GABA receptors is increased in young patients (Blue et al., 1999), and benzodiazepine binding to GABA_A_R is decreased in adult patients (Yamashita et al., 1998). Interestingly, a recent study found KCC2 levels decreased in cerebrospinal fluid (CSF) of Rett syndrome patients (Duarte et al., 2013). Although these studies in patients point to a general alteration in GABAergic signaling in Rett syndrome, whether this alteration entails decreased or increased transmission is not clear yet.

Experiments in MECP2 deficient mice also point to a general impairment in the GABAergic system, with contrasting results on whether GABAergic transmission is increased or decreased depending on the brain area analyzed. In particular, in postnatal ventro-lateral medulla decreased levels of α_2_ GABA_A_R subunit-expression and a decrease in amplitude and frequency of sIPSCs were described (Medrihan et al., 2008). Conversely, recordings from layer V pyramidal neurons of older MECP2 KO mice showed no change in mIPSCs (Dani et al., 2005). Moreover, MECP2 KO mice present altered expression of VGAT in the thalamus with decreased expression in the ventro-basal complex and increased expression in the reticular thalamic nucleus accompanied by frequencies of mIPSCs changing in the same direction (Zhang et al., 2010). Finally, a decrease in GABA release has been also described in MECP2 deficient mice (Medrihan et al., 2008; Jin et al., 2013).

A demonstration for a pivotal role for GABAergic system in Rett syndrome was provided by a study that deleted MECP2 exclusively in GABAergic neurons (Chao et al., 2010). In this mouse model, many of the Rett syndrome symptoms such as increased stereotypical behaviors, progressive motor and respiratory dysfunctions, alterations in social behavior and premature death were reproduced and the epileptic phenotype clearly emerged. Accordingly, MECP2 is a regulator of gene transcription highly expressed in GABAergic neurons (Akbarian et al., 2001), and its deletion causes a decrease in mRNA levels of GAD65, as well as in calbindin and calretinin, although an increase in parvalbumin (Durand et al., 2012). Moreover, one of the targets of MECP2 is GABRB3 (Leblanc and Fagiolini, 2011). Finally, also indirect findings in MECP2 deficient mice suggest a role for GABA in the pathology of this disorder. Indeed, dark rearing, an intervention which strongly affects GABAergic transmission (Benevento et al., 1995; Lee et al., 2006a), was able to rescue parvalbumin interneuron connectivity in the visual cortex and visual acuity of MECP2 KO mice (Durand et al., 2012). Of note, the respiratory alterations found in these mice were alleviated by treatment with benzodiazepine Midazolam, further suggesting the involvement of the GABAergic system in the general pathology of Rett syndrome disorder (Voituron and Hilaire, 2011). Yet, deletion of MECP2 exclusively in excitatory neurons has been recently shown to lead to epileptic phenotype and reduction of GABAergic synapses, indicating that MECP2 is also important in these cells (Zhang et al., 2014).

### Down syndrome

Down syndrome (DS) is a genetic condition caused by the trisomy of the twenty first chromosome and it is the most common genetic cause of intellectual disability accompanied in adulthood by Alzheimer-like pathology (Costa and Scott-McKean, 2013). Despite its relative frequency (i.e., 1 in 700 live births), the molecular mechanisms that mediate it are largely unknown. Nevertheless, several data from both patients and mouse models of DS support the notion that alterations in GABAergic signaling may underlie the cognitive component of this pathology (Kleschevnikov et al., 2004; Fernandez et al., 2007; Chakrabarti et al., 2010; Martinez-Cue et al., 2013). Indeed, GABA levels are reduced in the fetal frontal cortex (Whittle et al., 2007) and in the temporal lobes of children with DS (Smigielska-Kuzia et al., 2010). Conversely, results from postmortem adult brains are mostly unclear describing a decrease in the number of calbindin and parvalbumin interneurons in cortex, but a non-significant trend to reduced GABA in different brain regions, possibly because experiments were performed on brains from patients who had developed Alzheimer-like pathology (Reynolds and Warner, 1988; Kobayashi et al., 1990; Seidl et al., 2001). Additionally, a study with human neural progenitor cells found increased α_2_ and decreased α_5_ and β_3_ GABA_A_R-subunit expression (Bhattacharyya et al., 2009). Thus, as for Rett syndrome, studies on DS patients point to a general alteration in GABAergic signaling, although it is not clear yet whether this alteration is due to a decreased or an increased transmission.

To aid experimental investigation on DS, a number of mouse models have been generated, and among those, the Ts65Dn is the best characterized (Costa and Scott-McKean, 2013). Notably, a large body of work has pointed to a role for increased GABAergic transmission in these mice. First, a reorganization of inhibitory synapses of granule cells in the dentate gyrus and an increase in the number of calretinin, parvalbumin, somatostatin and calbindin interneurons were observed in the cortex (Belichenko et al., 2004; Chakrabarti et al., 2010; Perez-Cremades et al., 2010). Accordingly, GABAergic markers such as GAD65, GAD67, VGAT and gephyrin were elevated (Martinez-Cue et al., 2013). Moreover, GABA release (Begenisic et al., 2014) and evoked IPSCs of both GABA_A_ and GABA_B_ receptors were also increased (Kleschevnikov et al., 2012), with some authors finding increased GABAergic transmission particularly during the second postnatal week (Mitra et al., 2012). As Belichenko and coworkers found a decrease in β_2/3_ receptor at 3, but not at 8 months in the Ts65Dn model, this could indicate that not only the receptor distribution, but also its expression during certain developmental windows could be relevant to the disease (Belichenko et al., 2009b). Finally, Ts65Dn and Ts1Cje models of DS, showed decreased LTP and increased LTD in the hippocampus, which could be rescued by the use of GABA_A_ antagonists (Siarey et al., 1999; Kleschevnikov et al., 2004; Costa and Grybko, 2005; Belichenko et al., 2007, 2009a; Fernandez et al., 2007). At the behavioral level, a number of treatments aimed at decreasing GABAergic transmission have been attempted in mouse models of DS with the aim of rescuing cognitive deficits. In particular, Fernandez and colleagues treated Ts65Dn animals with several GABA_A_ antagonists (i.e., picrotoxin, bilobalide and pentylenetetrazole) and observed an improvement in declarative memory in the novel-object recognition and the spontaneous-alternation tasks (Fernandez et al., 2007). Interestingly, 2-week treatment with pentylenetetrazole maintained improved memory in the novel object recognition task up to 2 months after treatment discontinuation. Furthermore, an independent study showed that pentylenetetrazole was also able to rescue spatial memory in the Morris water maze in Ts65Dn mice (Rueda et al., 2008).

Unfortunately, chronic administration of GABA_A_ antagonists carries the risk of increasing seizure susceptibility. Thus, this pharmacological approach may not be readily translated into clinical practice because of the higher incidence of seizures that characterizes DS patients (and mouse models; Westmark et al., 2010; Rissman and Mobley, 2011). In the search of a better pharmacological approach that would avoid the risk of further increasing seizure susceptibility in DS, two independent groups specifically targeted the α_5_ subunit of the GABA_A_R. α_5_ subunit is highly expressed in the hippocampus (Sur et al., 1998) and authors predicted that its antagonization would provide enough disinhibition to rescue hippocampal-dependent memory. On the other hand, since α_5_ is not widely expressed in other brain regions, authors also predicted that risk of seizure induction would be less by a α_5_ subunit antagonist than by general GABA_A_R antagonists. In particular, Braudeau and coworkers found that treatment with an α_5_ inverse agonist rescued spatial memory as well as object-recognition memory in Ts65Dn mice (El Idrissi et al., 2005; Braudeau et al., 2011a,b; Potier et al., 2014). Moreover, recent findings describe that treatment with a negative allosteric modulator of α_5_ subunit-containing GABA_A_Rs rescues spatial memory, Schaffer collateral LTP, GAD65, GAD67, VGAT and gephyrin levels in Ts65Dn mice (Martinez-Cue et al., 2013). Interestingly, this treatment also increases cellular proliferation in the dentate gyrus (Martinez-Cue et al., 2013). This could indicate restored adult neurogenesis, which is also affected in DS models and partly responsible for the cognitive impairment (Rueda et al., 2005; Clark et al., 2006; Ishihara et al., 2010; Contestabile et al., 2013). Based on all of these studies, Hoffmann-La Roche has recently completed a Phase 1 and started a Phase 2 clinical trial (NCT01436955, NCT02024789, http://clinicaltrials.gov/) with an α_5_ inverse agonist aimed at measuring safety and cognitive improvement in DS patients, but no results have been posted yet.

Finally, also indirect evidence suggests that GABAergic treatments may ameliorate DS symptoms. For example, treating Ts65Dn pups with fluoxetine results in the normalization of a number of anatomical defects in the dentate gyrus-CA3 region of the hippocampus (Stagni et al., 2013), and treatment during adulthood rescues hippocampal adult neurogenesis (Clark et al., 2006). Since fluoxetine strongly and directly alters GABAergic hippocampal neurotransmission independently of its effect on amine reuptake systems (Mendez et al., 2012), it is tempting to speculate that fluoxetine effect on DS may be mediated by GABAergic transmission. Accordingly, a chronic treatment with fluoxetine normalizes GABA release and promotes recovery of cognitive impairment and hippocampal synaptic plasticity in adult Ts65Dn mice (Begenisic et al., 2014).

Thus, based on studies on animal models, treatment of DS with drugs targeting GABAergic transmission seems a promising venue. Nevertheless, in the context of finding GABAergic treatment for DS with reduced side effects, it will be essential to investigate the mechanisms involved in increased seizure prevalence in both humans and mouse models (Pueschel et al., 1991; Westmark et al., 2010).

### Schizophrenia

Schizophrenia is a neurodevelopmental disorder characterized by psychosis and cognitive deficits (Lewis et al., 2012). Multiple evidence shows that GABA plays a role in the development of schizophrenia by affecting mostly (but not only) cognitive aspects of the disorder (Ahn et al., 2011; Gonzalez-Burgos et al., 2011). Indeed, alterations in most domains of GABA-signaling have been described in schizophrenic patients. For example, variations in the GAD1 gene can increase risk of schizophrenia, and schizophrenic patients who carry a single nucleotide polymorphism in this gene have increased GAD25/GAD67 ratios in the hippocampus, suggesting that an immature GABAergic system is retained (Hyde et al., 2011). Moreover, GAD67, GAD65, and reelin (a protein mainly secreted by GABAergic interneurons) mRNAs and proteins are decreased in a number of brain regions, including the prefrontal cortex and cerebellum of schizophrenic patients (Guidotti et al., 2000; Fatemi et al., 2005; Hashimoto et al., 2008). These reductions are also observed in rodent models of schizophrenia. For example, chronic stimulation of D2 dopaminergic receptors reduces GAD67 expression (Schmidt and Mirnics, 2012), NMDA-receptor antagonism by phencyclidine (PCP) reduces mRNA levels of GAD65 and GAD67 (Bullock et al., 2009), isolation of rats leads to a decrease in reelin protein as well as number of reelin-positive cells in the dentate gyrus (Cassidy et al., 2010), and neonatal ventral hippocampus lesions decrease GAD67 mRNA in adult rats, an effect that can be counteracted by antipsychotic treatments (Lipska et al., 2003).

Specific alterations to GABAergic interneurons such as a decrease in the density of calbindin interneurons of the anterior cingulate cortex (Cotter et al., 2002) and a decrease in mRNA for markers of different interneuron subpopulations (i.e., somatostatin, parvalbumin and cholecystokinin) across different regions have been also described in schizophrenic patients (Hashimoto et al., 2008; Fung et al., 2010). Interestingly, parvalbumin downregulation is also common to genetic (Erbb4, DISC1) and non-genetic (PCP treatment, methylazoxymethanol administration during gestation, isolation) rodent and primate models (PCP-treatment; Cochran et al., 2003; Harte et al., 2007; Hikida et al., 2007; Morrow et al., 2007; Fisahn et al., 2009). In particular, deletion of Erbb4 from fast-spiking interneurons in mice leads to a decrease in GAD67 and parvalbumin protein levels, aberrant neuronal excitability and behavioral abnormalities (impairment in working memory, sociability and pre-pulse inhibition), consistent with a schizophrenic phenotype (Del Pino et al., 2013). Moreover, chandelier interneuron terminals in the prefrontal cortex of patients show a decreased GAT1 expression (Woo et al., 1998; Pierri et al., 1999), and GAT1 mRNA is decreased in cerebellum of PCP-treated rats with GAT1 positive chandelier interneuron cartridges decreased in the cortex of isolated rats (Bloomfield et al., 2008; Bullock et al., 2009). Taking advantage of this knowledge, Yu and colleagues explored the behavioral phenotype of GAT1 KO mice, and concluded it could be used as a model of schizophrenia (Yu et al., 2013). Although the above described results on GAD and on the different subpopulations of interneurons in schizophrenia would predict a net decrease in GABA production, measurements of total GABA in brains of patients by magnetic resonance spectroscopy (MRS) are conflicting, describing both a decrease and an increase (Goto et al., 2009; Ongur et al., 2010; Yoon et al., 2010).

Alterations in different GABA_A_R subunits were also reported in schizophrenic patients, including decrease in mRNA for α_1_, α_5_, β_2_, δ, and θ subunits and increase in α_2_ and ρ_2_, subunits (Volk et al., 2002; Hashimoto et al., 2008; Beneyto et al., 2011; Fatemi et al., 2013). On the other hand, increased mRNA levels of α_1_, α_6_, β_2_, β_3_, and δ, as well as GABA_A_R binding have been described in animal models (Endo et al., 2007; Bullock et al., 2009). A postmortem study showing increased GABA_A_R binding in the prefrontal cortex of schizophrenic patients further adds to the theory of disrupted GABAergic activity in this disorder (Benes et al., 1996). Thus, from this experimental evidence GABAergic transmission appears either increased or decreased in schizophrenic patients and animal models, depending on the brain area.

Interestingly, benzodiazepine treatment (alone or in combination with antipsychotics) has been used with mixed results for the treatment of anxiety and management of psychotic symptoms in schizophrenia. Nevertheless, 30–50% of patients respond favorably, possibly suggesting that GABA alterations are present in some patients, but not in others (Wolkowitz and Pickar, 1991). Data showing that antagonism of GABA_A_Rs by iomazenil treatment increases psychosis in only 50% of schizophrenics (and not in healthy controls) further strengthen this hypothesis (Ahn et al., 2011). To take advantage of this seeming therapeutic opportunity, Lewis and colleagues performed a first clinical trial using a benzodiazepine-like drug with specificity for receptors containing α_2_ and α_3_ subunits, thus avoiding α_1_ and α_5_, which are mostly involved in the side effects of conventional benzodiazepines. This study showed improved delayed memory in the in schizophrenic patients (Lewis et al., 2008). Nevertheless, a latter study did not find any improvement using the same compound in a larger sample of patients (Buchanan et al., 2011). As discussed above, the difference may rest in the fact that the impairment of the GABAergic signaling may be present only in a subpopulation of patients.

Finally, also factors that influence [Cl^−^]_i_ concentration have been implicated in schizophrenia. For instance, the SLC12A2 gene, which encodes for NKCC1, has been reported to be a susceptibility gene for schizophrenia (Potkin et al., 2009). Notably, Kim and colleagues found that an interaction between a single nucleotide polymorphism in the SLC12A2 gene and a single nucleotide polymorphism in the Disrupted in schizophrenia 1 (DISC1) gene increased risk for the development of schizophrenia (Kim et al., 2012). Moreover, KCC2 mRNA was decreased together with increased NKCC1/KCC2 ratios in hippocampus of patients (Hyde et al., 2011). Interestingly, the same group has recently reported differential expression of NKCC1 splice variants and an increase of a new transcript variant of the KCC2 gene in schizophrenia patients (Tao et al., 2012; Morita et al., 2014). Finally, although expression of NKCC1 and KCC2 mRNA was found unaltered in a study on prefrontal cortex of schizophrenic patients, the same study found increased expression of OXSR1 and WNK3 transcripts, which act posttranscriptionally to up-regulate NKCC1 and downregulate KCC2 activity (Arion and Lewis, 2011).

### Tourette syndrome

Tourette Syndrome (TS) is a neurodevelopmental disorder characterized by the presence of movement and vocalization tics. It is often comorbid with obsessive compulsive behavior, anxiety, attention deficit hyperactivity disorder and sleep disorders (Jankovic, 2001; McNaught and Mink, 2011). It also presents with a higher incidence of epilepsy (Williams et al., 2013). As for the other neurodevelopmental disorders described above, these symptoms suggest an altered neuronal excitatory/inhibitory ratio, with a possible implication of GABAergic signaling. Accordingly, postmortem staining of basal ganglia of TS patients revealed a decrease in the number of parvalbumin (PV) interneurons in the caudate and putamen as well as an increase in the same population in the globus pallidus pars interna (Kalanithi et al., 2005; Kataoka et al., 2010). Furthermore, a recent study on GABA_A_R binding in TS patients has shown a decreased in several regions (i.e., ventral caudate, putamen, nucleus acumbens and globus pallidus) and increase in others (i.e., substancia nigra, cerebellum and left periaductal gray; Lerner et al., 2012). Moreover, a recent genetic study found a correlation between severity of symptoms and increased blood expression of GABA_A_R subunit α_2–4_, β_1_, ρ_1_, and GABA_A_R-associated protein in patients (Tian et al., 2011). The same study also showed that genes encoding for the α_4_ and γ_1_ GABA_A_R subunits presented alternative splicing (Tian et al., 2011). Further strengthening the idea of an implication of the GABAergic system in TS is the fact that microinjections of GABA_A_ antagonists into the striatum of both mice and primates induce motor tics resembling those of Tourette patients (McCairn et al., 2009; Worbe et al., 2009; Bronfeld et al., 2013). Furthermore, pharmacological blockade of fast-spiking interneurons in the striatum is sufficient alone to elicit movement abnormalities also resembling those of TS (Gittis et al., 2011). Finally, there is some clinical evidence of GABAergic drugs being useful to control tics. In particular, clonazepam and levetiracetam have shown a modest profile in tic suppression, although more double-blind trials are needed to draw definitive conclusions (Shprecher and Kurlan, 2009; Martinez-Granero et al., 2010).

### Neurofibromatosis type 1

Neurofibromatosis type 1 is a genetic disorder caused by mutations in the NF1 gene. The phenotype is not homogeneous, and it includes presence of neurofibromas, cognitive impairment and increased epilepsy (Costa and Silva, 2002; Diggs-Andrews and Gutmann, 2013; Ostendorf et al., 2013). Increased hyperactivity, sleep disturbance, and emotional issues are also present (Johnson et al., 2005; Leschziner et al., 2013).

Both evidence in patients and studies in the NF1 KO mouse model of neurofibromatosis type 1 point to defects in GABAergic transmission, although with contrasting results. A recent study used MRS to measure total GABA in the visual cortex of NF1 patients and found that its level was decreased in comparison to controls (Violante et al., 2013). Conversely, work with NF1 mutant mice showed an increased GABA release as well as GABAergic transmission, and hypothesized them to be a cause for cognitive impairment (Costa et al., 2002; Cui et al., 2008; Shilyansky et al., 2010). In particular, NF1 KO mice showed an increase in the amplitude of evoked IPSPs, in the frequency of spontaneous IPSCs, and in the frequency of miniature IPSCs when measured in high potassium medium (Costa et al., 2002; Cui et al., 2008; Shilyansky et al., 2010). Cui and colleagues also observed that the increase in frequency of the miniature IPSCs was still present when the NF1 mutation was induced exclusively in interneurons, but not when it was only present in pyramidal neurons, again pointing to a key role of GABAergic transmission in the disease (Cui et al., 2008). Interestingly, NF1 KO mice also show deficits in CA1hippocampal LTP, spatial learning and working memory that can be reverted by the GABA_A_ antagonist picrotoxin (Costa et al., 2002; Cui et al., 2008; Shilyansky et al., 2010).

### Succinic semialdehyde dehydrohenase deficiency, tuberous sclerosis complex, angelman syndrome, Prader-Willi syndrome

Although less frequent and less studied, other neurodevelopmental disorders have also been related to defective GABAergic transmission.

Succinic semialdehyde dehydrohenase deficiency (SSDHD) is caused by a deficit of the GABA catabolitic enzyme succinic semialdehyde dehydrohenase and it thus results in an increase of GABA and γ-hydroxybutiryc acid (GHB, a by-product of GABA catabolism). The disorder courses with mental retardation, autistic-like behavior, hypotonia and seizures (Pearl et al., 2011; Vogel et al., 2013).

Tuberous sclerosis complex (TSC) is caused by loss of function of TSC1 and TSC2 genes, and courses with presence of cortical tubers, mental retardation, autistic-like behavior and epilepsy. Interestingly, studies of the tubers have shown decreased expression of GABA_A_R α_1_ subunit, decreased benzodiazepine-receptor binding and decreased expression of KCC2, as well as increased GABA levels and increased expression of NKCC1 (Talos et al., 2012). Interestingly, treatment with vigabatrin (an inhibitor of GABA-T) improves cognitive and behavioral issues and it is effective for controlling seizures in TSC (Jambaque et al., 2000).

Angelman and the closely related Prader-Willi Syndromes, are the result of deletion/methylation of chromosome region15q11–13. If the mutation is of maternal origin, it will give rise to Angelman Syndrome, whereas if the mutation is of paternal origin, the result will be Prader-Willi Syndrome (Zafeiriou et al., 2013). Interestingly, although considered distinct pathologies, both disorders share symptoms such as mental retardation and autism-like behaviors, and Angelman Syndrome patients are at higher risk of developing seizures (Buiting, 2010; Zafeiriou et al., 2013). Since the chromosomal region involved in these two disorders contains the genes of three GABA_A_R subunits (i.e., α_5,_ β_3,_ and γ_3_; Fiumara et al., 2010), this may indicate an involvement of the GABAergic system, as confirmed by some studies suggesting abnormal GABA metabolism in these disorders (Dhossche et al., 2005).

## GABAergic therapies: toward innovation and beyond

Neurodevelopmental disorders are a number of heterogeneous conditions that share a strong social and emotional impact on the lives of patients and their families. Although their etiology is very different and they are characterized by a variety of clinical pictures, they surprisingly share a large number of symptoms such as cognitive impairment, increased seizure susceptibility and sleep disturbance. Interestingly, an excitatory/inhibitory imbalance in neuronal activity -at least partly due to a disruption in the GABAergic system- also appears as a common feature to most of these pathologies.

From a historical point of view, research aimed to develop therapeutic strategies mostly addressing either GABA_A_R function or GABA metabolism, possibly because it simply represented the more intuitive approach. For example, in a number of studies researchers directly targeted GABA signaling by treatment of animal models with GABA_A_ agonists (i.e., autism, Fragile X, Rett syndrome; Olmos-Serrano et al., 2011; Voituron and Hilaire, 2011; Han et al., 2012) or antagonists (i.e., Down syndrome and Neurofibromatosis type I; Fernandez et al., 2007; Cui et al., 2008). Although encouraging results indicate improvement of both cognitive and non-cognitive symptoms in these models, it is important to be cautious when translating data from animal models to humans. For example, certain apparent discrepancies between animal models of the diseases and patients such as those described for Down syndrome, Rett syndrome, schizophrenia, and Neurofibromatosis type 1 have to be properly addressed, possibly through parallel studies in animals and humans by the same researchers with similar experimental approaches, when achievable.

Since most discussed developmental disorders present increased risk of epileptic seizures, adequate controls for seizure activity should be performed. Furthermore, since GABA is a very widespread neurotransmitter acting both at the CNS and PNS levels and in a number of periphery organs (Roberts and Krause, 1982; Watanabe et al., 2002), treatments with GABA_A_ antagonists could lead to excessive disinhibition through the blocking of both phasic and shunting inhibition and treatment with GABA_A_ agonists can lead to undesired side effects such as drowsiness and muscle relaxation (Rudolph et al., 1999; Griebel and Holmes, 2013). Thus, issues on the potentials of translating experimental findings into the clinical practice, forced researchers to find more subtle ways to modulate GABAergic transmission. The discovery that different GABA_A_R subunits finely modulate GABAergic responses in physiology (e.g., tonic vs. phasic inhibition) inspired researchers to target specific subunits in neurodevelopmental disorders to reduce side effects of GABA_A_ergic pharmacological treatments (e.g., the specific antagonists of the α_5_ subunit in the Ts65Dn animal model of Down syndrome to recover cognitive deficits without pro-epileptic side effects; Braudeau et al., 2011b; Martinez-Cue et al., 2013). In addition, GABA_A_ agonists selective for the α_2,3_ subunits are being researched as potential anxiolytic/analgesic drugs that lack the sedative effects, which are mainly mediated by α_1_ (Mohler, 2011), and these drugs are also being tested for schizophrenia (Lewis et al., 2008; Buchanan et al., 2011; Mohler, 2011). Moreover, it has been recently argued that drugs acting on the GABA_A_ δ subunit can aid the treatment of Fragile X (Hagerman et al., 2009; Olmos-Serrano et al., 2011; Heulens et al., 2012). Given the latter encouraging results, it is possible that future therapies -if aimed at targeting GABA_A_R subunits specifically affected in the diverse diseases- will present even further reduced side effects by restoring proper brain function with no effect on other “non-diseased” receptors. However, further research focusing on alteration of specific GABA_A_ subunits in each different neurodevelopmental disorder and further research focusing on developing drugs that selectively act on these specific subunits are a prerequisite before treatment implementation (Mohler, 2011).

The discovery that action of NKCC1/KCC2 could indirectly modulate GABAergic transmission opened new avenue for therapeutic research readably translatable into clinical trials. First, high expression of NKCC1 in the neonatal brain was suggested to be a concomitant cause aggravating epilepsy in infants (Dzhala et al., 2005). Then, modulation of GABAergic transmission trough variation in the activity of cation/Cl^−^ cotransporters was described under particular physiological conditions (e.g., delivery and adult neurogenesis) and in neurodevelopmental disorders (e.g., Fragile X, Rett syndrome, schizophrenia). As NKCC1 inhibitor bumetanide is an FDA-approved loop diuretic extensively used in the past with very mild side effects (Kahle and Staley, 2008), clinicians were readily allowed to perform clinical trials on the use of bumetanide to treat infant and adult epilepsy, and more recently autism in young patients with encouraging results (Kahle et al., 2009; Lemonnier and Ben-Ari, 2010; Lemonnier et al., 2012; Eftekhari et al., 2013; Hadjikhani et al., 2013). As neurodevelopmental disorders are characterized by increased susceptibility to seizures, diuretic treatment seems a very promising approach also because loop diuretics have been clinically tested in the past as antiepileptics by a mechanism proposed to be independent from GABAergic transmission (Maa et al., 2011). Indeed, bumetanide decreases cell volume and increases the extracellular space, both of which contribute to decrease neuronal excitability and synchrony of neuronal network by means of the so called “ephaptic effect,” namely a synapse-independent form of propagation of electrical signals (Haglund and Hochman, 2005; Maa et al., 2011; Hochman, 2012). Moreover, action on cation chloride cotransporters is predicted to be a subtler approach with milder side effects than direct action on GABA_A_Rs by agonists/antagonists as shunting inhibition (which critically depends on the number of open GABA_A_Rs) results only slightly affected by small changes in [Cl^−^]_i_. Nevertheless, as bumetanide does not pass well the blood-brain barrier (BBB) in mice, more lipophylic bumetanide prodrugs are being developed to address this issue, while avoiding the diuretic effect due to bumetanide action on NKCC2, an isoform present in the kidney. Some of these drugs have recently been tested in rodents and seem to have better brain penetration (Loscher et al., 2013; Tollner et al., 2014). On the other hand, a completely new compound (CLP257) was very recently described to restore Cl^−^ balance by increasing KCC2 expression at the plasma membrane and it was able to rescue nociceptive responses in a rat model of neuropathic pain, providing a new and efficient tool to tackle hyperexcitability in the SNC (Gagnon et al., 2013).

Finally, also environmental enrichment (EE), which exerts a profound influence over GABAergic transmission (Cancedda et al., 2004; Sale et al., 2010) results in behavioral, synaptic and connectivity rescues in models of neurodevelopmental disorders (Restivo et al., 2005; Nithianantharajah and Hannan, 2006; Lonetti et al., 2010; Begenisic et al., 2011; Durand et al., 2012; Hannan, 2014), and could thus be considered a possible therapeutic approach. In particular, EE rescues spatial memory and dentate gyrus LTP as well as neuronal proliferation in Ts65Dn animals (Begenisic et al., 2011; Chakrabarti et al., 2011). Moreover, cortical LTP, anxiety behavior, motor coordination and spatial learning could be rescued in MECP2 null mice by EE (Kondo et al., 2008; Nag et al., 2009; Kerr et al., 2010; Lonetti et al., 2010) with an indication that the inhibitory GABAergic system could be preferentially responsible for the effect (Boggio et al., 2010; Lonetti et al., 2010). Furthermore, EE restored environment exploration and dendritic branching/length in Fmr1KO mice (Restivo et al., 2005), and it was able to ameliorate some symptoms in a genetic model of schizophrenia (McOmish et al., 2008). Finally, behavioral impairments such as nociception, motor coordination, pre-pulse inhibition, stereotyped behavior, grooming, memory deficits, anxiety, social deficits were rescued in a number of mice models of ASD reared in an EE (Schneider et al., 2006; Lacaria et al., 2012; Reynolds et al., 2013). Although EE does seem ideal as a non-invasive treatment, there is however controversy as to how well it will translate to the human setting, given that some of the effects that are sometimes reported in environmentally enriched animals could be just considered as the rescue of the environmental deprivation of the standard housing conditions of laboratory animals (Nithianantharajah and Hannan, 2006; Sale et al., 2014). Nevertheless, the first recent attempts to translate EE into the humans setting have proven useful for the treatment of autism (Woo and Leon, 2013).

## Concluding remarks

In conclusion, we reviewed that modulation of GABAergic transmission plays a pivotal role in regulating brain activity under physiological conditions during development, and disruption of GABAergic signaling is a hallmark of many neurodevelopmental disorders. Thus, correcting GABA signaling in pathological conditions could reinstate physiological level of brain function in a pathological background. On the other hand, because of the complex role for GABA in regulating physiological neuronal activity during development and in adulthood, careful consideration should be paid in the treatment of neurodevelopmental disorders by GABAergic therapies to decrease side effects. We propose that further dissection of the molecular mechanisms in place for the regulation of brain development under physiological conditions may inspire researchers to address GABAergic transmission with a subtle and physiological approach also in neurodevelopmental disorders. Moreover, a detailed study of the specific defects in GABAergic transmission in the different neurodevelopmental disorders must be achieved before being able to design the best therapeutic strategies. Also, it will be compulsory to perform thorough research on humans as data in animal models are at times not paralleled by findings in humans. In the near future, studies with human-derived induced pluripotent stem (iPS) cells will greatly aid to resolve this issue, by unraveling the fine molecular pathways affected in each disorder in humans. Indeed, cell lines derived from human patients are already available for mutations related to ASD such as SHANK3, as well as for Fragile X, Rett syndrome, Down syndrome, and schizophrenia (Marchetto et al., 2010; Urbach et al., 2010; Brennand et al., 2011; Li et al., 2012). Thus, basic research on the role of GABAergic transmission during physiological and pathological brain development is a fundamental prerequisite in the design of innovative pharmacological treatments with reduced side effects.

### Conflict of interest statement

Laura Cancedda is on the Provisional Application: US 61/919,195, 2013. Modulators of Intracellular Chloride Concentration For Treating An Intellectual Disability.
